# RAGE Knockout Mitigates Diet-Induced Obesity and Metabolic Disruption

**DOI:** 10.3390/metabo15080524

**Published:** 2025-08-02

**Authors:** Isabelle L. Palmer, Genevieve Parker, Alden T. Chiu, Colson G. Beus, Ethan P. Evans, Jack H. Radford, Cameron R. Braithwaite, Ryan D. van Slooten, Elijah T. Cooper-Leavitt, Zachary E. Moore, Derek M. Clarke, R. Ryley Parrish, Juan A. Arroyo, Paul R. Reynolds, Benjamin T. Bikman

**Affiliations:** Department of Cell Biology and Physiology, Brigham Young University, Provo, UT 84602, USA

**Keywords:** advanced glycation end products, mitochondria, adipose, obesity

## Abstract

**Background/Objectives:** The receptor for advanced glycation end products (RAGEs) has been implicated in obesity and metabolic dysfunction. However, its precise role in diet-induced obesity remains unclear. **Methods:** In this study, we investigated the metabolic consequences of RAGE knockout (RAGE KO) in mice subjected to a Western diet (WD). **Results:** Our findings demonstrate that RAGE KO mice remained significantly leaner than their wild-type (WT) counterparts when fed a WD, exhibiting reduced body weight gain and smaller adipocyte size. Indirect calorimetry revealed that RAGE KO mice had increased oxygen consumption and locomotor activity compared to WT mice, indicating enhanced energy expenditure. Mitochondrial respiration assays indicated significantly greater oxygen consumption in RAGE KO animals. Additionally, systemic inflammation markers, such as TNF-α, were significantly lower in RAGE KO mice when fed a WD, indicating a reduction in diet-induced inflammatory responses. **Conclusions:** These findings suggest that RAGE plays a key role in metabolic homeostasis, and its deletion confers resistance to obesity and metabolic disruption induced by a Western diet. Targeting RAGE may provide a novel therapeutic approach for combating obesity and related metabolic disorders.

## 1. Introduction

Obesity and its associated metabolic disorders represent a growing global health crisis, contributing to the escalating prevalence of type 2 diabetes, cardiovascular disease, and other chronic conditions. The etiology of obesity is multifactorial, involving genetic predisposition, environmental influences, and hormonal regulation, which collectively disrupt adipocyte biology and energy homeostasis. A hallmark of obesity is the chronic, low-grade inflammation that develops in expanding adipose tissue—referred to as meta-inflammation—which plays a central role in the pathogenesis of obesity-induced metabolic dysfunction [1].

A major contributor to this inflammatory response is the receptor for advanced glycation end products (RAGEs), a pattern recognition receptor implicated in a range of chronic diseases including diabetes, atherosclerosis, and neurodegeneration [2,3,4]. RAGE activation occurs through binding with various endogenous ligands such as advanced glycation end products (AGEs), high-mobility group box 1 (HMGB1), and S100/calgranulin proteins [5,6], triggering sustained inflammatory signaling cascades via nuclear factor-kappa B (NF-κB) and mitogen-activated protein kinase (MAPK) pathways [7]. This prolonged activation amplifies the production of pro-inflammatory cytokines like tumor necrosis factor-alpha (TNF-α) and various interleukins, contributing to insulin resistance and systemic metabolic impairment [6,8].

Animal studies have shown that mice lacking RAGE exhibit reduced adipose tissue inflammation, enhanced insulin sensitivity, and improved energy expenditure in models of diet-induced obesity [9,10]. These effects are thought to involve not only inflammatory suppression but also improved mitochondrial function. Mitochondria, central to cellular energy metabolism, are known to be disrupted in obesity and metabolic syndrome [11], while increased mitochondrial biogenesis and efficiency are associated with protection against weight gain and insulin resistance [12]. Importantly, RAGE signaling has been linked to impaired mitochondrial respiration and oxidative phosphorylation [13], suggesting that RAGE may serve as a nexus between inflammation and mitochondrial dysfunction in obesity.

Despite this growing understanding, the specific mechanisms by which RAGE influences mitochondrial bioenergetics in adipose tissue remain incompletely understood. The present study aims to address this gap by characterizing the metabolic and mitochondrial consequences of RAGE deletion in a murine model of diet-induced obesity. We hypothesize that RAGE knockout (KO) mice will demonstrate reduced adiposity, improved energy expenditure, and enhanced mitochondrial function compared to wild-type controls. Through integrated analyses of adipose morphology, mitochondrial respiration, and systemic inflammation, this study seeks to elucidate the role of RAGE in regulating energy metabolism under conditions of nutritional excess and identify potential therapeutic avenues for obesity-related metabolic disease.

## 2. Methods

### 2.1. Animals and Tissue Preparation

All animal procedures were approved by the Institutional Animal Care and Use Committee (IACUC) and conducted in accordance with the National Institutes of Health guidelines for the care and use of laboratory animals. C57BL/6 mice were obtained from Jackson Laboratories (Bar Harbor, ME, USA). Male and female wild-type (WT) and RAGE knockout (RAGE KO) mice were housed in temperature- and humidity-controlled conditions under a 12 h light/dark cycle with ad libitum food and water access. At 6 weeks of age, mice were randomly assigned to either a standard diet (SD; 10% kcal from fat) or a Western diet (WD; 45% kcal from fat, D12266B, Research Diets, New Brunswick, NJ, USA; 8 per group) for a period of 12 weeks. Although both sexes were used, no statistically significant sex differences were observed across groups. Therefore, results are presented combined, consistent with recommendations to avoid overstratification in the absence of effect.

### 2.2. Body Composition and Adipocyte Morphology

Body weight was measured weekly. Perirenal adipose tissue was collected post-euthanasia, then weighed and fixed in 4% paraformaldehyde. Adipocyte size and morphology were determined via hematoxylin and eosin (H&E; Sigma-Aldrich, Burlington, MA, USA) staining and analyzed using ImageJ Version 1.5 software (Bethesda, MD, USA).

### 2.3. Metabolic and Energy Expenditure Assessments

Indirect calorimetry was performed using a Comprehensive Lab Animal Monitoring System (CLAMS; Columbus Instruments, Columbus, OH, USA) to measure oxygen consumption (VO_2_), carbon dioxide production (VCO_2_), and locomotor activity over a 48 h period. Respiratory exchange ratio (RER) was calculated as VCO_2_/VO_2_ to assess substrate utilization. Locomotor activity was determined by infrared beam breaks over 10 min intervals. Food intake was continuously monitored throughout the metabolic assessment.

### 2.4. Mitochondrial Respiration Analysis

Mitochondrial function in adipose tissue was assessed using high-resolution respirometry (Oroboros O2k; Oroboros Instruments, Innsbruck, Austria), as we have performed previously [14]. Briefly, freshly extracted and prepared perirenal adipose tissue was placed in respirometer chambers and given time to calibrate. Following this, a simple substrate-based protocol was utilized. To determine a complex I- and II-mediated respiration, samples were simultaneously treated with glutamate (10 mM), malate (2 mM), and succinate (10 mM; GMS). Once stable, adenosine diphosphate (ADP; 2.5 mM) was added to determine respiration rates during oxidative phosphorylation.

### 2.5. Inflammatory Marker Analysis

Plasma levels of tumor necrosis factor-alpha (TNF-α) were quantified using enzyme-linked immunosorbent assay (ELISA; Thermo Fisher Scientific, Waltham, MA, USA) according to the manufacturer’s instructions.

### 2.6. Statistical Analysis

All statistical analyses were performed using GraphPad Prism 10 (GraphPad Software, Boston, MA, USA). Data are expressed as mean ± SEM. Two-way ANOVA was used to assess the main effects of genotype and diet, as well as their interaction. Bonferroni post hoc tests were applied when significant effects were found. Interaction terms are now specified in the figure legends. Statistical significance was set at *p* < 0.05.

## 3. Results

### 3.1. RAGE KO Mice Are Protected from Fat Gain on Western Diet

**Figure 1:**
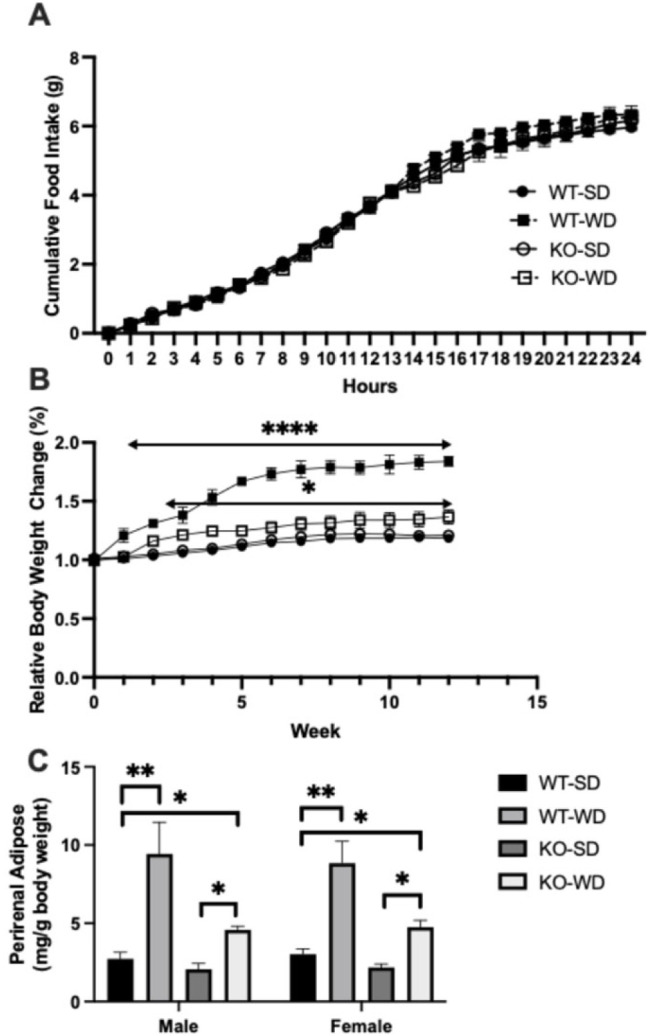
Cumulative food intake over 24 h in wild-type (WT) and RAGE KO (KO) mice on standard chow diet (SD) and Western diet (WD) (**A**). Relative body weight changes over 12 weeks in WT and RAGE KO mice on standard (SD) or Western diet (WD) (**B**), with perirenal adipose in the same groups (**C**) expressed per unit total body mass (Two-way ANOVA with Bonferroni post hoc testing; *n* = 7; **** *p* < 0.0001, ** *p* < 0.01, * *p* < 0.05. Unless indicated, vs. WT-SD).

During a distinct (24 h) period, we monitored cumulative food intake across all groups, namely wild-type (WT) and RAGE (KO) mice on standard (SD) and western (WD) diets. There were no differences in food consumption across any group (Figure 1A). Despite this, there were substantial differences in relative weight gain (Figure 1B), with the WT-WD animals gaining substantially more weight than the other groups, including the RAGE KO group on the WD (KO-WD) as well. The KO-WD group gained more than the SD groups, albeit to a less degree. Importantly, at least some of these weight differences were due to changes in fat mass, as similar trends were noted with higher perirenal fat mass (Figure 1C; expressed per unit body mass) in the WT-WD group.

### 3.2. Adipocyte Size Differs Across Conditions

**Figure 2:**
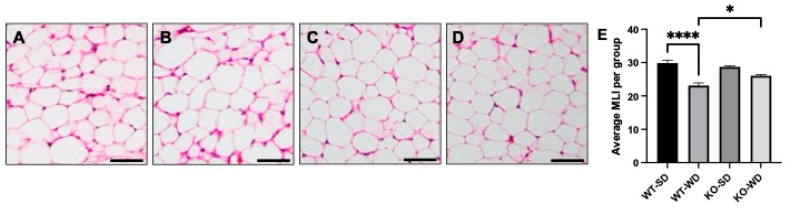
Adipocyte morphology and mean linear intercept (MLI) in perirenal adipose tissue in wild-type (WT) and RAGE KO (KO) mice on standard chow diet (SD) and Western diet (WD). WT-SD (**A**), WT-WD (**B**), KO-SD (**C**), KO-WD (**D**), all quantified via MLI (**E**). Scale bars represent 50 µm (Two-way ANOVA with Bonferroni post hoc testing; *n* = 5; **** *p* < 0.0001, * *p* < 0.05).

With the observed changes in adipose mass across the conditions, we sought to understand the degree of adipocyte hypertrophy via hematoxylin and eosin (H&E) staining. Adipocytes from WT-WD mice (Figure 2B) were considerably larger than the SD-fed counterpart (Figure 2A), as measured via mean linear intercept (MLI; 2E). Furthermore, the WT-WD MLI was significantly lower (reflecting larger adipocyte diameter) than the KO-WD (Figure 2D), suggesting a reduced adipocyte size in the RAGE KO animals.

### 3.3. Increased Energy Expenditure in RAGE KO Mice

**Figure 3:**
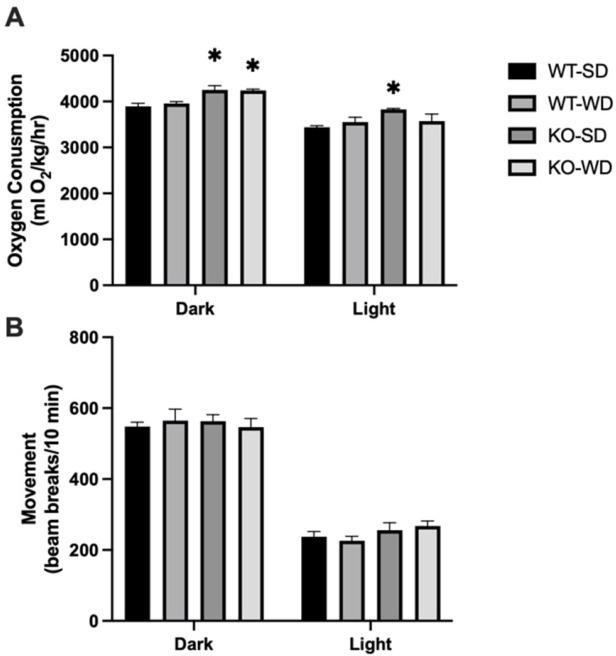
Metabolic rate (**A**) and movement (**B**) were measured via oxygen consumption in mice across phenotype and diet (Two-way ANOVA with Bonferroni post hoc testing; *n* = 4; * *p* < 0.05 vs. SD).

The apparent incongruency between the similar food consumption yet varied adiposity suggested a difference in metabolic rate, which we tested via indirect calorimetry. Our results demonstrated that RAGE KO mice had significantly higher oxygen consumption compared to WT mice (Figure 3A) in the dark phase, with light-phase differences only in the KO-SD group. Interestingly, this appeared to occur independently from physical activity, as movement did not differ between groups (Figure 3B).

### 3.4. Altered Mitochondrial Bioenergetics in RAGE KO Mice

**Figure 4:**
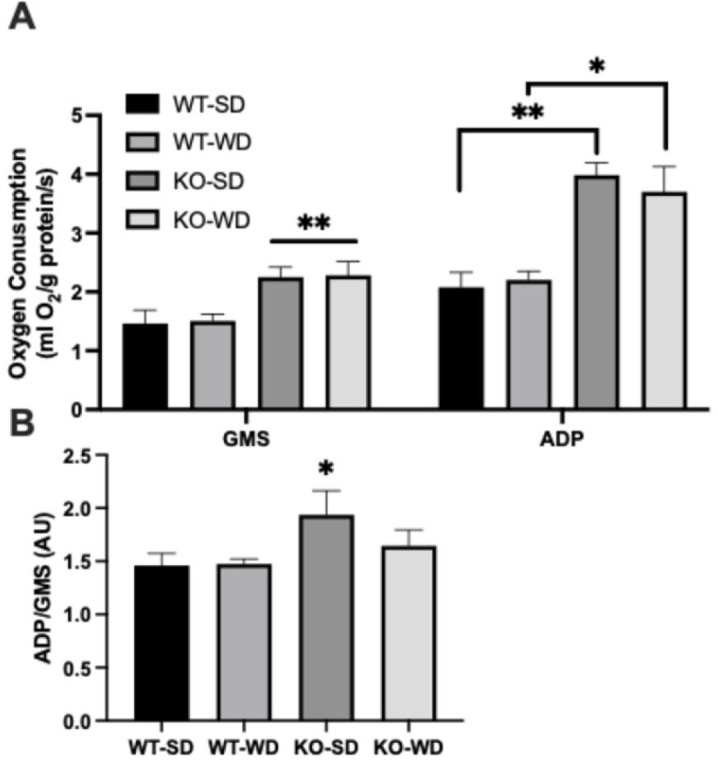
Oxygen consumption (**A**) from permeabilized perirenal adipose tissue was measured in two states, with glutamate + malate + succinate (GMS) and ADP. ADP/GMS was determined as an indicator of respiratory control (**B**) (Two-way ANOVA with Bonferroni post hoc testing; *n* = 6; ** *p* < 0.01; * *p* < 0.05).

Mitochondrial respiration analysis revealed that perirenal adipose tissue from RAGE KO mice exhibited significantly higher oxygen consumption rates under both complex I + II (glutamate + malate + succinate; GMS) substrates alone and with ADP (Figure 4A) in both dietary conditions. Interestingly, when we compared this with the effect of oxidative phosphorylation-mediated respiration as a function of GMS-supported respiration, only the KO-SD group was significantly different (Figure 4B). Although the ADP/GMS ratio does not represent a classical respiratory control ratio (RCR), it serves here as an index of ADP-stimulated respiration relative to substrate-supported respiration in intact tissue, consistent with prior usage in the bioenergetic profiling of complex tissues.

### 3.5. Reduced Systemic Inflammation in RAGE KO Mice

**Figure 5:**
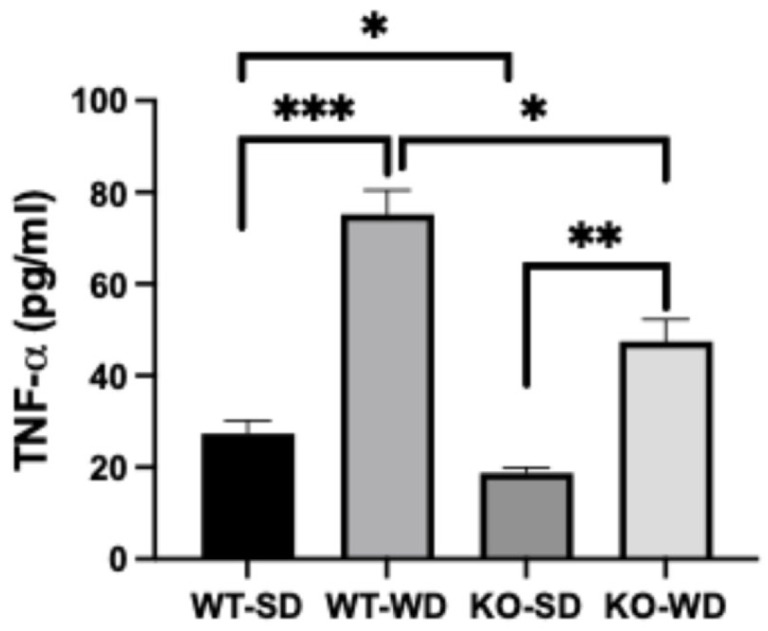
Plasma TNF-α concentrations in WT and RAGE KO mice under SD and WD (Two-way ANOVA with Bonferroni post hoc testing; *n* = 6; *** *p* < 0.001; ** *p* < 0.01; * *p* < 0.05).

Hypertrophic adipocytes contribute heavily to systemic inflammation via adipose-derived pro-inflammatory cytokines. Plasma TNF-α levels were elevated substantially in the animals fed a Western diet vs. standard diet, regardless of genotype (Figure 5). However, the RAGE KO animals, in turn, consistently had lower levels of TNF-α compared with the WT animals.

## 4. Discussion

This study highlights the essential role of the receptor for advanced glycation end products (RAGEs) in mediating the adverse metabolic consequences of a high-fat, high-carbohydrate Western diet (WD). Across myriad assessments, RAGE knockout (KO) mice demonstrated a significantly healthier metabolic profile than their wild-type (WT) counterparts, including reduced adiposity, robust mitochondrial respiration, increased energy expenditure, and suppressed systemic inflammation—despite equivalent caloric intake. Collectively, these findings reinforce the concept that RAGE contributes directly to energy imbalance and metabolic dysfunction.

### 4.1. RAGE Deletion Confers Protection Against Obesity and Inflammation

RAGE KO mice gained significantly less weight and accumulated considerably less perirenal adipose tissue while consuming a WD compared with WT mice. This was likely at least partly due to reductions in adipocyte size, as RAGE KO mice consistently had smaller adipocyte diameter. Far more than simply indicating obesity, adipocyte diameter itself is a highly relevant variable in metabolic health, insofar as hypertrophy of adipocytes leads to the robust production and release of pro-inflammatory cytokines [1]. These data align with previous reports that RAGE promotes adipocyte inflammation and hypertrophy [10]. Moreover, these observations suggest that RAGE deletion alters adipose tissue remodeling, favoring a healthier phenotype, supporting the findings of others [9,10]. Our findings indicate that removing this signaling input preserves adipose tissue function and limits excessive expansion in response to dietary stress.

As mentioned, obesity-induced inflammation is a hallmark of metabolic dysfunction, particularly hypertrophic adipocytes. Importantly, our data show that RAGE KO mice exhibited significantly lower plasma levels of TNF-α, a key pro-inflammatory cytokine elevated in obesity. This reduction occurred despite WD feeding and modest fat gain, suggesting that RAGE signaling is necessary to sustain the chronic low-grade inflammation typically associated with nutrient excess. Moreover, RAGE is known to activate NF-κB and MAPK pathways, both of which drive persistent inflammatory cytokine production [15]. By disrupting this axis, RAGE deletion likely dampens immune activation in adipose tissue and the systemic circulation. Furthermore, this anti-inflammatory effect may indirectly enhance mitochondrial function, as inflammatory cytokines are known to impair mitochondrial bioenergetics [16].

### 4.2. Enhanced Energy Expenditure and Mitochondrial Bioenergetics in RAGE KO Mice

Where our work is novel compared with previous reports is our characterization of energy expenditure and adipose mitochondrial bioenergetics. Collectively, our findings help reconcile the thermodynamics. After all, the leaner phenotype in RAGE KO mice occurred without any reduction in food intake or increase in physical activity. Instead, indirect calorimetry revealed a substantial rise in oxygen consumption, indicating elevated basal metabolic rate. This suggests that RAGE deletion promotes intrinsic elevation in energy dissipation. Moreover, the dissociation between physical activity and oxygen consumption in these mice underscores a shift in basal metabolic programming, rather than behaviorally driven energy usage. This shift likely reflects changes at the mitochondrial level.

Indeed, consistent with previous reports that RAGE impairs mitochondrial dynamics and substrate oxidation [13], our study found that RAGE KO mice exhibit increased oxidative phosphorylation capacity in adipose tissue. Mitochondrial respiration rates were significantly elevated, both at baseline and under ADP-stimulated conditions. Furthermore, this increase in mitochondrial respiration likely contributes to the elevated energy expenditure observed in vivo [17]. Greater mitochondrial respiration suggests greater metabolic rate and substrate utilization—including fatty acids—while reducing the accumulation of intracellular mediators of insulin resistance.

In addition, mitochondrial dysfunction is a well-established contributor to obesity and type 2 diabetes [18]. Thus, the increased mitochondrial capacity in RAGE KO mice may underlie the broader metabolic benefits observed in this model, including enhanced insulin sensitivity and protection against adipose tissue expansion.

### 4.3. Future Direction

While our findings clearly demonstrate the protective metabolic effects of RAGE deletion in diet-induced obesity, including novel findings on mitochondrial function, future work is needed to further elucidate the cellular and molecular underpinnings of these observations, particularly within adipose tissue. A key avenue of interest is the inflammatory landscape of adipose depots. Although we observed reductions in systemic TNF-α levels, the extent and nature of immune cell infiltration into adipose tissue remain uncharacterized. Future studies will incorporate immunohistochemistry (IHC) techniques to quantify macrophage infiltration and polarization status (i.e., M1 vs. M2 phenotypes), which are critical determinants of adipose tissue inflammation and systemic insulin sensitivity.

Moreover, the AGE/RAGE axis is a known mediator of oxidative stress and cellular dysfunction. While we demonstrate improved mitochondrial bioenergetics in RAGE KO mice, additional analyses are warranted to explore the degree of oxidative stress at the tissue level. Future experiments will include assays for reactive oxygen species (ROS) production and antioxidant capacity within adipose tissue to determine whether RAGE deletion also confers resistance to oxidative insults. These assessments could involve quantification of lipid peroxidation products (e.g., MDA), antioxidant enzymes (e.g., SOD, catalase), and mitochondrial oxidative damage markers. Gene expression profiling will also be expanded to include transcripts relevant to AGE/RAGE-mediated pathways, including levels of pro-inflammatory cytokines, oxidative stress-responsive genes, and apoptosis-related markers to assess how RAGE influences broader cellular stress and survival responses in adipose tissue. Further, given RAGE’s known link to cellular stress, future work will evaluate mitochondrial dynamics—including fusion and fission regulators MFN2 and DRP1—as well as mitophagy pathways (e.g., PINK1, Parkin) that may be altered in response to chronic nutrient excess. This will allow us to determine whether RAGE deletion modifies susceptibility to AGE-induced damage via transcriptional reprogramming.

### 4.4. Translational Potential and Therapeutic Implications

Given the alarming rise in global obesity rates and their associated comorbidities, identifying molecular targets like RAGE is of high clinical relevance. While genetic deletion of RAGE offers mechanistic insights, it is important to note that this model does not replicate the context or specificity of pharmacologic inhibition in humans. Therefore, translational claims must be made with appropriate caution. Nevertheless, our findings suggest that RAGE inhibition could offer myriad benefits, including protection against obesity, reducing inflammatory burden, and maintaining mitochondrial function. Not surprisingly, several RAGE antagonists are currently in development, and our data provide additional mechanistic rationale for their application in metabolic disease contexts.

Beyond genetic deletion, emerging evidence suggests that RAGE expression in adipose tissue is also modifiable by environmental and lifestyle factors, particularly glycemic control. Hyperglycemia has been shown to upregulate RAGE expression in adipocytes through the production of reactive oxygen species and accumulation of advanced glycation end products (AGEs), which bind and activate RAGE, perpetuating inflammation and insulin resistance [19]. This suggests that dietary or pharmacologic strategies aimed at reducing postprandial glucose excursions could attenuate RAGE expression and its downstream metabolic consequences. For example, low-glycemic diets, regular physical activity, and insulin-sensitizing therapies such as metformin may confer metabolic benefits in part by disrupting the AGE-RAGE signaling axis.

These insights underscore the potential for lifestyle interventions to complement pharmacologic approaches in targeting RAGE activity. Given our finding that RAGE knockout confers resistance to diet-induced adiposity and inflammation, it is plausible that reducing RAGE expression through modifiable behaviors could offer similar protection. Future studies should investigate whether glycemic control and antioxidant strategies can downregulate adipose RAGE expression and mimic the beneficial metabolic phenotype observed in RAGE KO models. Such approaches could offer accessible, non-invasive means to mitigate obesity-related metabolic dysfunction by modulating a key inflammatory pathway. Furthermore, we will need to examine whether pharmacological RAGE inhibition replicates these effects in human models of obesity and whether tissue-specific RAGE deletion reveals differential metabolic outcomes. Additionally, exploring interactions between RAGE and other metabolic regulators may uncover synergistic targets to combat diet-induced obesity and related cardiometabolic complications.

This study has several limitations. First, the modest sample size limits the statistical power to detect subtle metabolic differences, especially between sexes. Second, while RAGE KO models reveal important mechanistic roles, they do not fully reflect tissue-specific contributions or the effects of partial pharmacologic inhibition. Lastly, while our mitochondrial findings are consistent with enhanced oxidative function, further mechanistic work is needed to confirm whether observed changes stem from altered biogenesis, dynamics, or mitophagy.

## 5. Conclusions

This study establishes the receptor for advanced glycation end products (RAGEs) as a central regulator of metabolic disruption in response to a Western diet, revealing that RAGE knockout (KO) mice are protected from excessive adiposity, adipocyte hypertrophy, systemic inflammation, and mitochondrial dysfunction. Despite equivalent caloric intake and activity levels, RAGE KO mice exhibited increased energy expenditure and enhanced mitochondrial respiration, highlighting a metabolic reprogramming that resists nutrient excess. These findings underscore RAGE’s role at the intersection of inflammatory and metabolic signaling and suggest that inhibiting RAGE may offer a promising therapeutic avenue to mitigate obesity and its related comorbidities through both pharmacologic and lifestyle-based interventions.

## Data Availability

Data scans be made available by contacting the corresponding author.
